# Bridging the gap between decomposition theory and forensic research on postmortem interval

**DOI:** 10.1007/s00414-023-03060-8

**Published:** 2023-07-25

**Authors:** Blake M. Dawson, Maiken Ueland, David O. Carter, Donna Mclntyre, Philip S. Barton

**Affiliations:** 1 School of Environmental and Rural Science, University of New England, Armidale, NSW 2350 Australia; 2 Centre for Forensic Science, School of Mathematical and Physical Sciences, University of Technology Sydney, Sydney, NSW 2007 Australia; 3 Forensic Sciences Unit, School of Natural Sciences & Mathematics, Chaminade University of Honolulu, Honolulu, HI 96822 USA; 4 Graduate Research School, Federation University, Mount Helen, Ballarat, VIC 3350 Australia; 5 Future Regions Research Centre, Federation University, Mount Helen, Ballarat, VIC 3350 Australia; 6 School of Life and Environmental Sciences, Deakin University, Geelong, VIC 3216 Australia

**Keywords:** Necrobiome, Forensic science, Entomology, Ecology, Microbiome, Investigation, Decay, Taphonomy

## Abstract

Knowledge of the decomposition of vertebrate animals has advanced considerably in recent years and revealed complex interactions among biological and environmental factors that affect rates of decay. Yet this complexity remains to be fully incorporated into research or models of the postmortem interval (PMI). We suggest there is both opportunity and a need to use recent advances in decomposition theory to guide forensic research and its applications to understanding the PMI. Here we synthesise knowledge of the biological and environmental factors driving variation in decomposition and the acknowledged limitations among current models of the PMI. To guide improvement in this area, we introduce a conceptual framework that highlights the multiple interdependencies affecting decay rates throughout the decomposition process. Our framework reinforces the need for a multidisciplinary approach to PMI research, and calls for an adaptive research cycle that aims to reduce uncertainty in PMI estimates via experimentation, modelling, and validation.

## Introduction

The decomposition of organic matter is a fundamental but complex process that is influenced by multiple biotic and abiotic variables [1, 2]. These variables act in concert to determine rates of decay and biomass loss [3–5]. One application of this knowledge is to medicolegal investigations into the time of death [6], where estimations of the postmortem interval (PMI) may be important [5, 7]. However, current models for estimating PMI, or other approaches to measuring time-since-death, lack generality or ignore many of the complexities of decomposition [5, 8–10]. For example, taphonomic approaches may ignore insect activity, while insect-based approaches may ignore the importance of microbes or the role of larger vertebrate scavengers [11, 12]. Other intrinsic attributes of a deceased individual, such as body mass or pharmaceutical history, may further complicate matters. By not considering these potential sources of variation in decomposition, there is a real risk that estimates of the PMI will not be useful due to the large potential for error. Here we suggest that decomposition theory and concepts developed in the biological and ecological disciplines offer a way to overcome the current limitations to PMI estimates. This theory shows that decomposition is an inherently multidisciplinary process and that current models of PMI stem largely from disciplinary perspectives. We believe this is one primary reason why models of the PMI have generally performed poorly and why a new multidisciplinary approach is needed. We introduce a conceptual framework that can guide research on the PMI by showing, for the first time, the many interdependencies among key drivers of variation of decomposition. Our framework is based on a broad synthesis of the decomposition literature spanning ecological and forensic disciplines. Our hope with this paper is for readers to consider the complexities behind decomposition in their own research, and to consider how these complexities might inform casework or inspire new collaborations with decomposition scientists.

## Decomposition and the postmortem interval

After death, the remains of vertebrates (including humans) undergo decomposition and gradual breakdown until all tissues have disintegrated, digested, or been consumed by decomposer microorganisms and scavenger animals [13, 14]. Knowledge of the decomposition process has informed forensic and medicolegal investigation practices, including information relating to the PMI [6]. A PMI can be estimated as the minimum and/or maximum amount of time an individual has potentially been deceased [6, 15]. Estimation of the PMI, however, is complicated by the natural variability in the decomposition process [16, 17] and the effects of many environmental and biological factors on changes to human remains over time [9, 18–22]. In a forensic context, factors can include criminal activity such as wrapping, concealment, or burial, or other factors like scavenging by animals or preservation environments, for example. Accurate estimation of the PMI therefore depends on detailed knowledge of the decomposition process and scene findings [6, 23]. Current methods do not necessarily incorporate all key factors, leaving room for improvements in current models and techniques used to estimate the PMI [15].

Research on PMI estimation has generally focused on a narrow disciplinary perspective. Although this research is helpful to forensic casework, a narrow view can risk missing the complexities of decomposition and could lead to error in PMI estimation. For example, recent attention has been given to the appropriate use of pigs or other animals as analogues for human cadavers in taphonomic and entomological based research, yet the potential microbial community differences have mostly been ignored to date [19, 24–26]. There is a need, therefore, to continue development of PMI methods and models and incorporate the growing knowledge of the complexities of decomposition. This need is echoed by calls for greater standardisation and repeatability of methods used in decomposition research [23, 27, 28]. There have been few attempts to integrate the known drivers of decomposition into a multidisciplinary framework that accurately captures their effects and interactions. We suggest that by examining decomposition through a multidisciplinary lens, it is possible to view the many sources of variation in decomposition and find areas to improve future PMI research.

## Past models of PMI have not yet performed well

Although clinical based models of PMI estimation perform reasonably well during early stages of decomposition, other methods are required beyond 72 h [29]. However, there are no current models of mid- or late-stage decomposition that perform well following empirical validation and testing across a range of circumstances. Previous attempts at developing a ‘universal model’, therefore, have failed to deliver with investigators suggesting inadequate accounting of different variables affecting decomposition rate [10]. Vass [30] developed a model of PMI for surface remains built from measures of temperature, moisture, and an estimate of soft tissue decomposition stage. Cockle and Bell [8] attempted to verify this model, but found it was not supported, and suggested decomposition is more complex than previously thought [8]. Maile and Inoue [5] also tested the model and noted some limited success, but also highlighted limitations related to climate and observer subjectivity [5]. A new approach to PMI estimation is therefore needed that can account for variation brought about by different groups of decomposers, climate, and intrinsic human characteristics.

## A multidisciplinary approach to decomposition is needed to reflect complexity and interdependencies—an integrated framework to guide PMI research

The key challenge in decomposition research is quantifying and accounting for all the sources of variation that exist in the decomposition process. One key source of variability that has received considerable attention is the community of decomposer organisms and their interactions that forms the ‘necrobiome’. The necrobiome provides an important theoretical foundation for this multidisciplinary framework to integrate decomposition theory with estimation of the PMI. It places the biological and ecological functions and processes driven by microbes, insects, and vertebrates [1, 13, 14, 31] into a framework that includes their interactions with abiotic conditions, soil, and the surrounding ecosystem [1]. A key advancement stemming from the necrobiome concept is the links it reveals between different decomposer groups. For example, the primacy of microbes in decomposition has long been appreciated [32, 33], but their central role in shaping waves of decomposer colonisation of remains, and aspects of their behaviour, is becoming ever clearer [34–36]. Identification of such interdependencies allows for new investigations into the role of decomposer interactions [11].

**Fig. 1:**
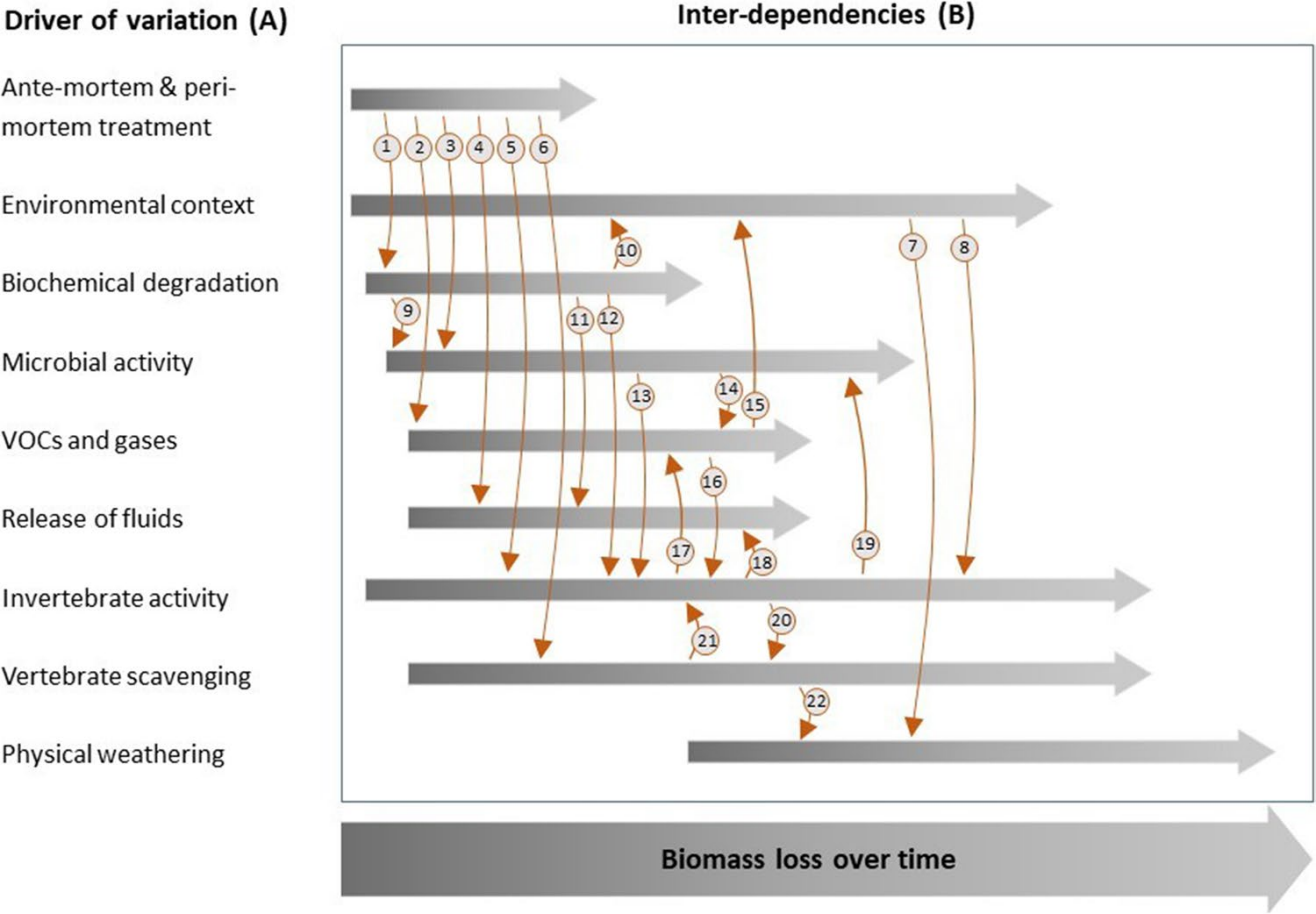
A multidisciplinary approach to the development of models to estimate the postmortem interval must reflect the complexities of decomposition. This could be achieved by recognising the numerous physicochemical, biological, and ecological drivers of variation (**A**) that are inter-related in ways that affect the rate and pattern of mass loss and decay of cadavers over time (**B**). Numbers on the arrows represent example mechanisms described in the peer-reviewed literature that link the drivers of variation. A list of mechanisms and references is provided in Table 1

The interconnected nature of decomposition processes presents a major roadblock to creating effective and flexible models for estimating the PMI. We suggest this roadblock can be overcome by exploring ways to incorporate the biological complexity being revealed by modern decomposition science into new models of the PMI. To facilitate this, we introduce a multidisciplinary framework (Fig. 1) that draws attention to the drivers of variation in decomposition and their multiple interdependencies. By bringing these multiple components and drivers of variation together, the full complexity of decomposition can be observed.

These drivers may be environmental, physicochemical, biological, or ecological, and their complex interdependencies mean a multidisciplinary approach is critical. For example, gases and volatile organic compounds (VOCs) emitted as the result of initial and subsequently changing microbial activity are shaped by the deceased individual’s background and environmental context, and this in turn will strongly affect the colonisation patterns of insects or olfaction-dependent vertebrate scavengers [11]. Exploration of such relationships might require expertise from pathologists, chemists, microbiologists, entomologists, and ecologists. The net effect of these drivers of variation is gradual mass loss via physical (dispersal directly into the local environment) and/or trophic (consumption) pathways, and which will alter the physical appearance of the cadaver. However, the importance of these drivers and their interactions will vary as decomposition proceeds over time. This means processes occurring during the early stages of decay (fresh/bloat stages) will be different from processes occurring later in decomposition (active/advanced/skeletonization stages). To develop more robust models of the PMI, our framework can assist with identifying what drivers of variation (A) are relevant during a particular phase of decomposition, and consider manipulating their interdependencies (B) via suitable experimental designs and treatments (Fig. 1).

During the first 24–48 h of decomposition, several PMI measures derived from different drivers of variation, such as rigor mortis (biochemical degradation), can be employed to accurately estimate a PMI [56]. During this time, only a few drivers of variation need to be considered, particularly peri-mortem treatment (Table 1). For example, microbial activity is directly influenced by peri-mortem treatment of an individual, such as chemotherapy or other chemical treatments, which may alter the microbiome, and subsequent decomposition progress [57]. The complexity of decomposition increases as decomposition progresses (i.e. active decay) as new drivers of variation are introduced, while the previous drivers are still relevant and active. Due to this added complexity, current PMI measures are often less reliable than those used in early decay [58]. For example, larval development rates are a common PMI measure, yet their accuracy may be impacted by several drivers of variation [59]. Insects locate remains using highly specialised chemoreceptors that detect decomposition VOCs, which are produced by microbial activity and biochemical degradation [50]. If these decomposition VOCs are altered, then the arrival time of primary colonising insects may be delayed; therefore, a minimum PMI derived from larval data could be drastically different from the actual time of death. Currently, some interdependencies are incorporated into larval development models (e.g. temperature), but the inclusion of more interdependencies should be considered (e.g. microbial activity), which may result in more accurate and reliable PMI measures that reduce the amount of unknown variation in decomposition. Table 1 lists all current interdependencies that have been identified and highlights example effects of each driver of variation.

**Table 1:** Examples of mechanisms linking sources of variation in the decomposition process displayed in Fig. 1

Link	Source of variation	Driver impacted	Example mechanism	Reference
1	Ante- & peri-mortem treatment	Biochemical degradation	Differences in human and animal protein degradation profiles	[37]
2		VOCs and gases	Odour profiles differed between cadaver types	[38]
3		Microbial activity	Soil microbial profiles differed under bison and elk carrion	[39]
4		Release of fluids	Cadaver type affected soil nutrient profiles	[26]
5		Invertebrate activity	Cadaver type influenced invertebrate succession patterns	[25]
6		Vertebrate scavenging	Scavengers preferred human cadavers over non-human carrion	[40]
7	Environmental context	Physical weathering	Skeletal weathering was affected by shade	[41]
8		Invertebrate activity	Shade reduced insect diversity and abundance	[42]
9	Biochemical degradation	Microbial activity	Input of proteins into soil, which increased microbial turnover of labile N compounds	[43]
10		Environmental context	Increased concentration of grave soil nutrients around cadaver	[44]
11		Release of fluids	Decomposition of tissue led to N enrichment of fluids over time	[45]
12		Invertebrate activity	Nutrient and moisture transfer to insect consumers and soil	[46]
13	Microbial activity	Invertebrate activity	Microbe-laden marine carrion had fewer invertebrate scavengers than fresh carrion	[47]
14		VOCs and gases	Bacteria community altered VOCs emitted	[48]
15	VOCs and gases	Environmental context	Contrasting VOCs in soil compared to air	[49]
16		Invertebrate activity	Specific VOCs attract flies	[50]
17	Invertebrate activity	VOCs and gases	Insects contribute to VOCs emitted	[51]
18		Release of fluids	Larvae feeding reduced nutrients entering soil	[52]
19		Microbial activity	Insect exclusion led to altered microbial functional activity	[33]
20		Vertebrate scavenging	Invertebrates outcompete vertebrate facultative scavengers	[53]
21	Vertebrate scavenging	Invertebrate activity	Presence of vertebrates reduced blowfly fecundity	[54]
22		Physical weathering	Rapid bone exposure and damage by vultures	[55]

## Multidisciplinary framework in action

Below we give three case studies of research on decomposition that highlight the ways in which multidisciplinary approaches can reveal important drivers of variation in the decomposition process. When viewed through the lens of our framework, it becomes clear how these case studies all share the important feature of generating new knowledge that a single disciplinary focus could not.

### Case study 1. Examining multiple drivers of pig decomposition in Hawaii

**Fig. 2:**
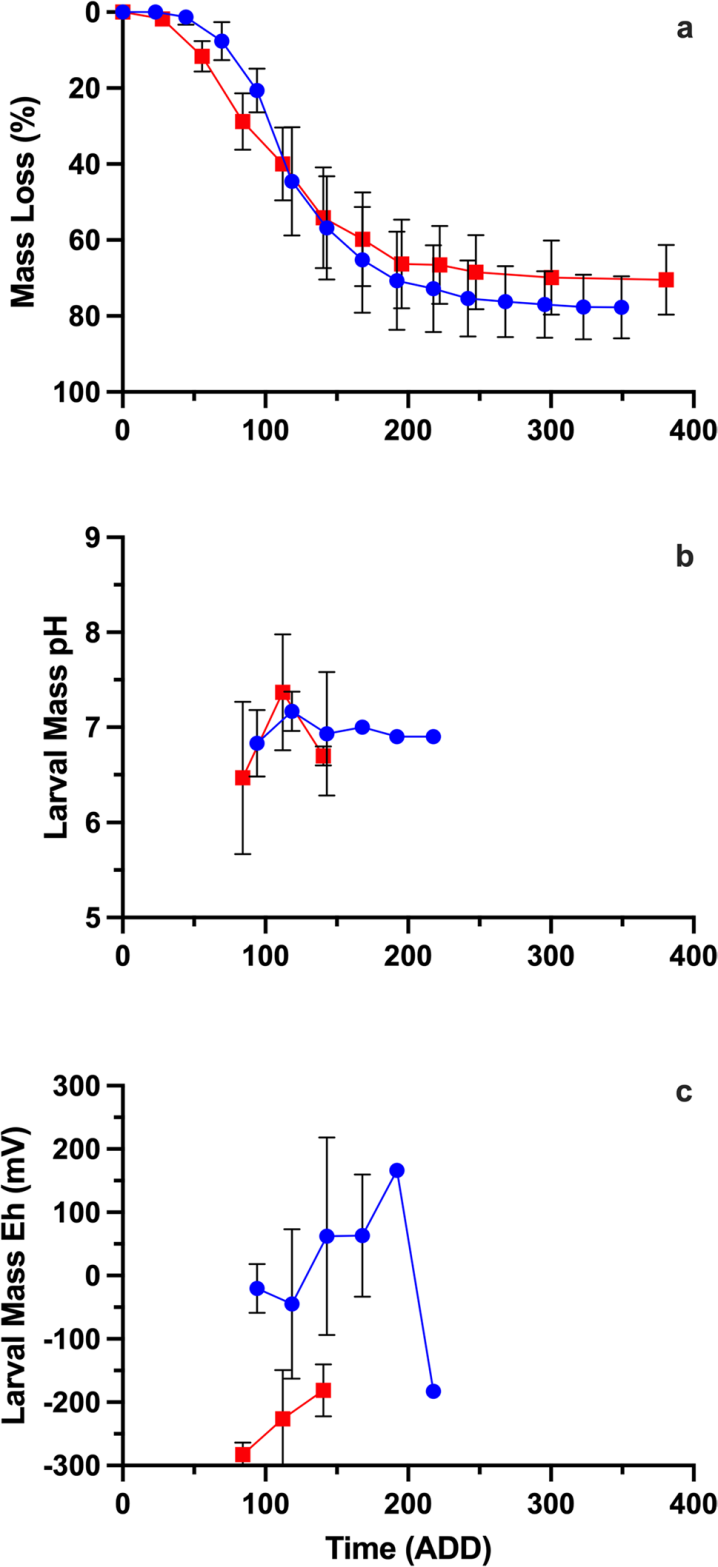
Mean ± s.e. mass loss (**a**), fly larval mass pH (**b**), and fly larval mass oxidation reduction potential (milliVolts) (**c**) of pig carcasses over 14 days (~350 accumulated degree days) of decomposition in a tropical climate during the summer (red) and winter (blue) where *n* = 3

Research is being conducted on Oahu, Hawaii, to understand the influence of seasonal variation on the decomposition of pig carcasses and associated decomposer activity. Decomposition studies, each using 3 pig carcasses, have been conducted to compare carcass decomposition between spring, summer, autumn, and winter. The measurement of gross decomposition is complemented by measurements of larval mass pH and oxidation reduction potential (Eh). The purpose of this research is to identify seasonal trends in decomposition processes while investigating the role of microbes and insects in these processes. For example, results from these studies show that carcass mass loss and larval mass pH can be relatively consistent between seasons (Fig. 2a). However, the appearance and duration of larval masses can differ between seasons, as can other chemical properties like Eh (Fig. 2b, c). The Eh of carcasses appear to play a particularly significant role in the structure of microbial communities associated with carcass decomposition [60]. Results from these studies demonstrate the importance of considering multiple drivers of decomposition because a range of environmental and postmortem characteristics can be associated with a similar rate and pattern of decomposition in this tropical setting. However, these relationships might vary in other locations that differ in temperature, humidity, and decomposer diversity. The development of a multidisciplinary framework is crucial to understanding the variation in taphonomy across space and time.

### Case study 2. Identifying microbial and insect interdependencies and their link to decomposition rate

**Fig. 3:**
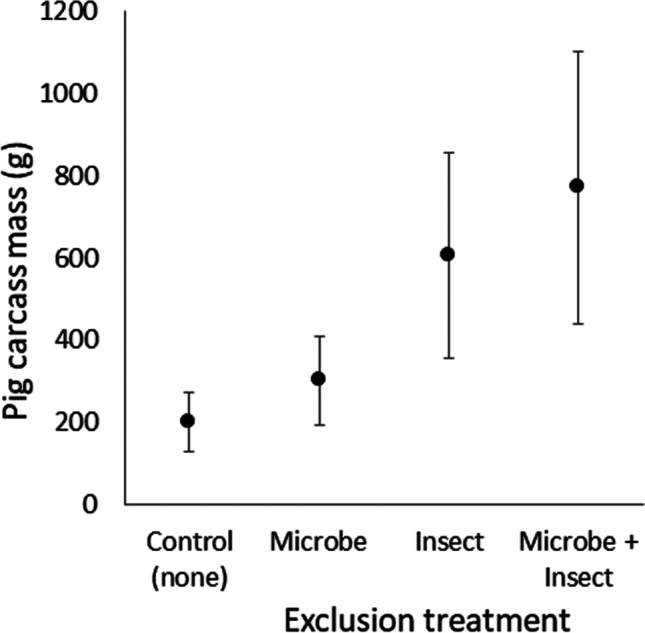
Mean ± s.e. mass of pig carcasses remaining after 23 days of decomposition. Pigs were exposed to four different treatments: control = full access by decomposers, insect = exclusion of insects, microbe = disinfectant applied to external surface, insect microbe = both insect exclusion and microbial disinfectant (*n* = 5 per treatment)

Research is being conducted in Australia to investigate interacting drivers of decomposition of pig carcasses. An experiment has been designed to expose 20 small pig carcasses to various combinations of insect exclusion or microbiome perturbation via the novel introduction of an antiseptic (chlorhexidine). The purpose of this research is to quantify the relative importance of insects and microbes in the decomposition process, particularly with regard to how this might affect changing interpretation or potential estimates of the postmortem interval. Results from the study showed a significant difference in mass loss among the experimental treatments after 23 days of decomposition (Fig. 3). The greatest mass loss (most rapid decay) occurred among carcasses with a fully intact necrobiome (controls), and the least mass loss (slowest decay) occurred on carcasses with both insects and microbes excluded. Further results also suggested a delay in the pre-appearance interval and oviposition of blowflies on pigs with the disturbed microbiome. Results from this experiment demonstrate the importance of considering multiple drivers of decomposition and their potential interaction when assessing decomposition.

### Case study 3. Investigating the effect of chemotherapy on multiple aspects of human decomposition

**Fig. 4:**
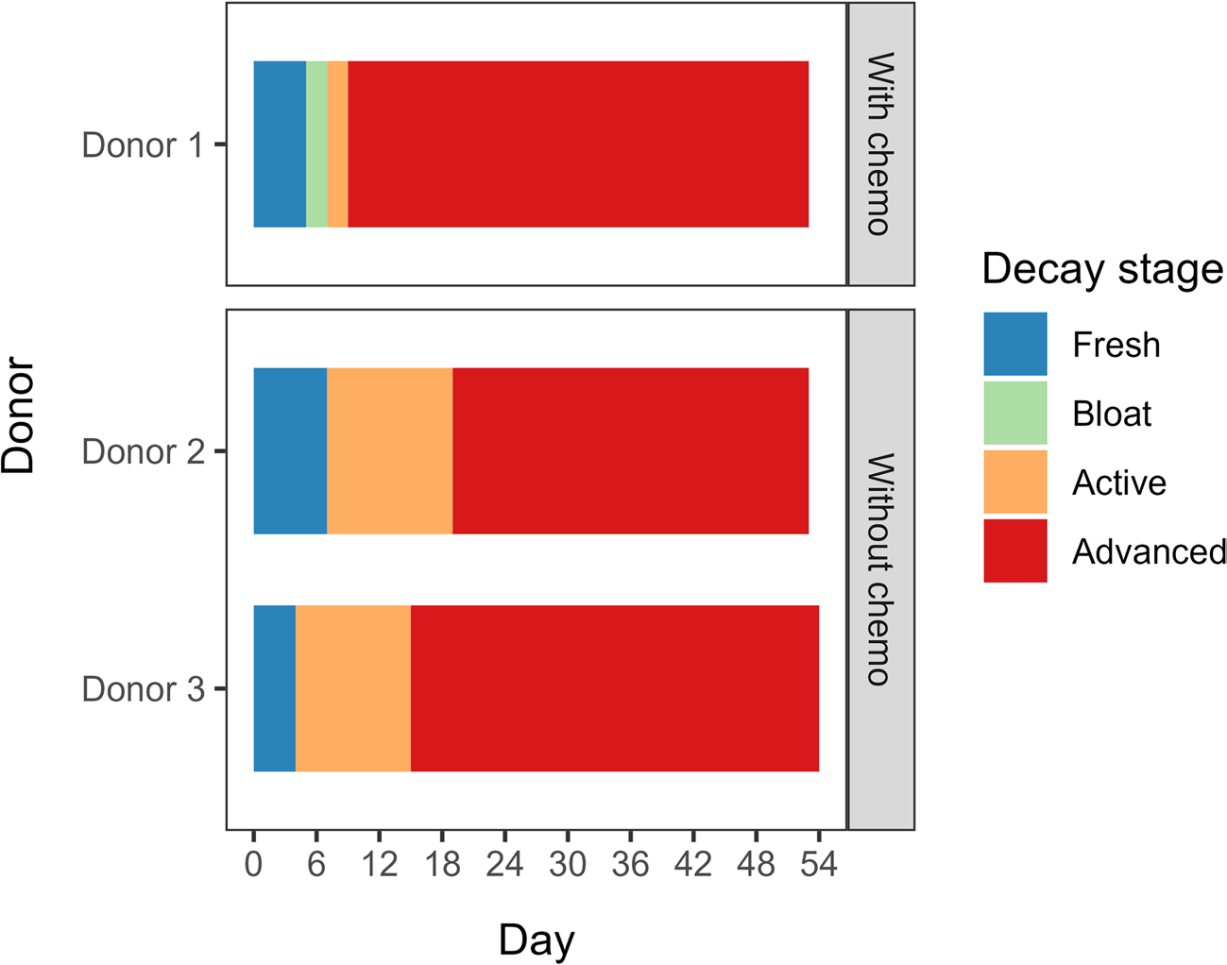
Visual stages of decomposition of the three donors (donor 1, donor 2, and donor 3) over the course of the experiment

**Fig. 5:**
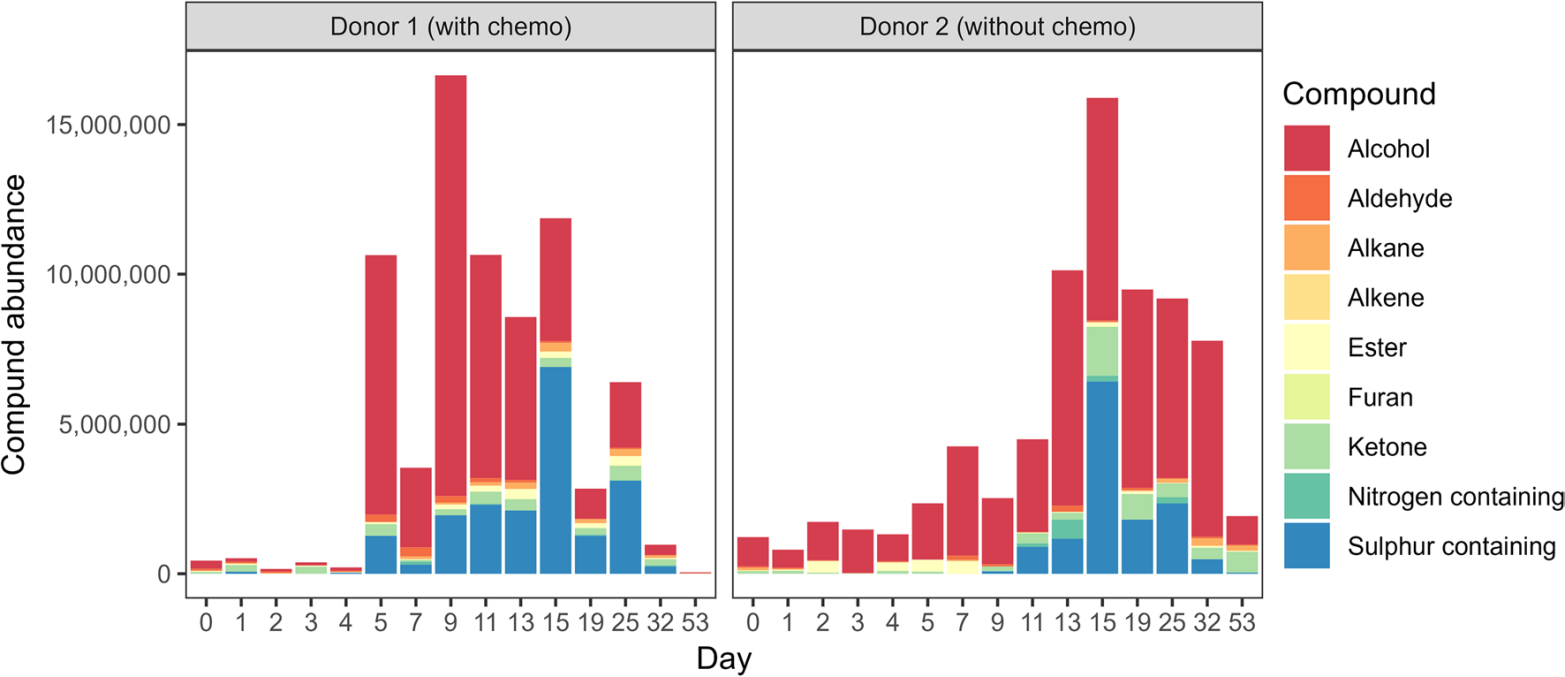
Abundance of chemical compound classes detected for donor 1 and donor 2

A study conducted at a human taphonomy facility in Australia investigated the effect of chemotherapy prior to death on the decomposition process. A donor (donor 1) who had significant cancer treatment prior to death was compared to two donors (donor 2 and donor 3) without any chemotherapy placed in the same month. Throughout the decomposition process, visual changes in decomposition (TBS) were monitored, insects were observed, and volatile organic compounds (VOCs) of donor 1 and donor 2 were collected. Donor 1 had limited insect activity, particularly around the head relative to the other donors. Another key difference observed was the limited time donor 1 spent in the active decay stage, where most of the breakdown occurs. Instead, the donor progressed almost immediately into an advanced stage where little activity (insect and mass loss) and decay was observed (Fig. 4). The visual decomposition of donor 1 halted. The volatile profiles between the two donors showed that the same compound groups were present; however, the abundance and occurrence differed (Fig. 5). This was particularly evident in the initial days where almost no VOCs were detected from donor 1, which likely explains the lack of fly activity and in turn, little breakdown occurring. The ante-mortem treatment of donor 1 had a direct impact on volatiles, and may have either directly or indirectly (altered VOCs) impacted insect activity, and therefore altered the entire decomposition process.

## Next steps for PMI research and experiments

Our multidisciplinary framework provides the foundation for new hypotheses to be identified and tested. This will enhance our knowledge of sources of variation in decomposition, and help to build new compound models of PMI that capture a wider range of decomposition variation, which can then be validated using experiments and applied to forensic casework.

To begin with, the foundation of any new model will be built on knowledge of the relationships and interdependencies identified here in this framework (Fig. 1; Table 1). These drivers of variation and any new relationships or interdependencies which may be contributing to variation in decomposition need to be quantified and tested. These relationships can be quantified by controlled experiments, isolating, and combining specific interdependencies. For example, to assess the relationship between insects and microbes, controls must be used that exclude other drivers.

Once these relationships have been identified and quantified, new models can be constructed that improve existing PMI measures, or created from entirely new measures. Additional complexity can be added to the models by incorporating other abiotic factors which have a known effect on the PMI measure. Importantly, these models would be regionally specific due to the large amount of variation that exists between localities, but as refinement continues, the models may be spatially upscaled once more variation is accounted for. In some localities, decomposition rates and factors impacting decomposition may be similar despite being geographically separated due to similar climates and conditions (e.g. consider *x* factors if in *y* localities).

The new models can be further validated using high replication and rigorous controls in a range of contexts to fully understand strengths and weaknesses of the models/measures, and the sources of variation. This is widely accepted in the ecological sciences but can be challenging in forensic research where replication of cadavers is difficult. Therefore, these high replicated experiments will likely need to be conducted on animal models, and eventually validated on human cadavers before any data or models can be used in forensic casework for PMI estimation. In some instances, correction factors will need to be applied to ensure past research on animal models can be appropriately applied to human cadavers to ensure previously conducted research does not need to be redone.

**Fig. 6:**
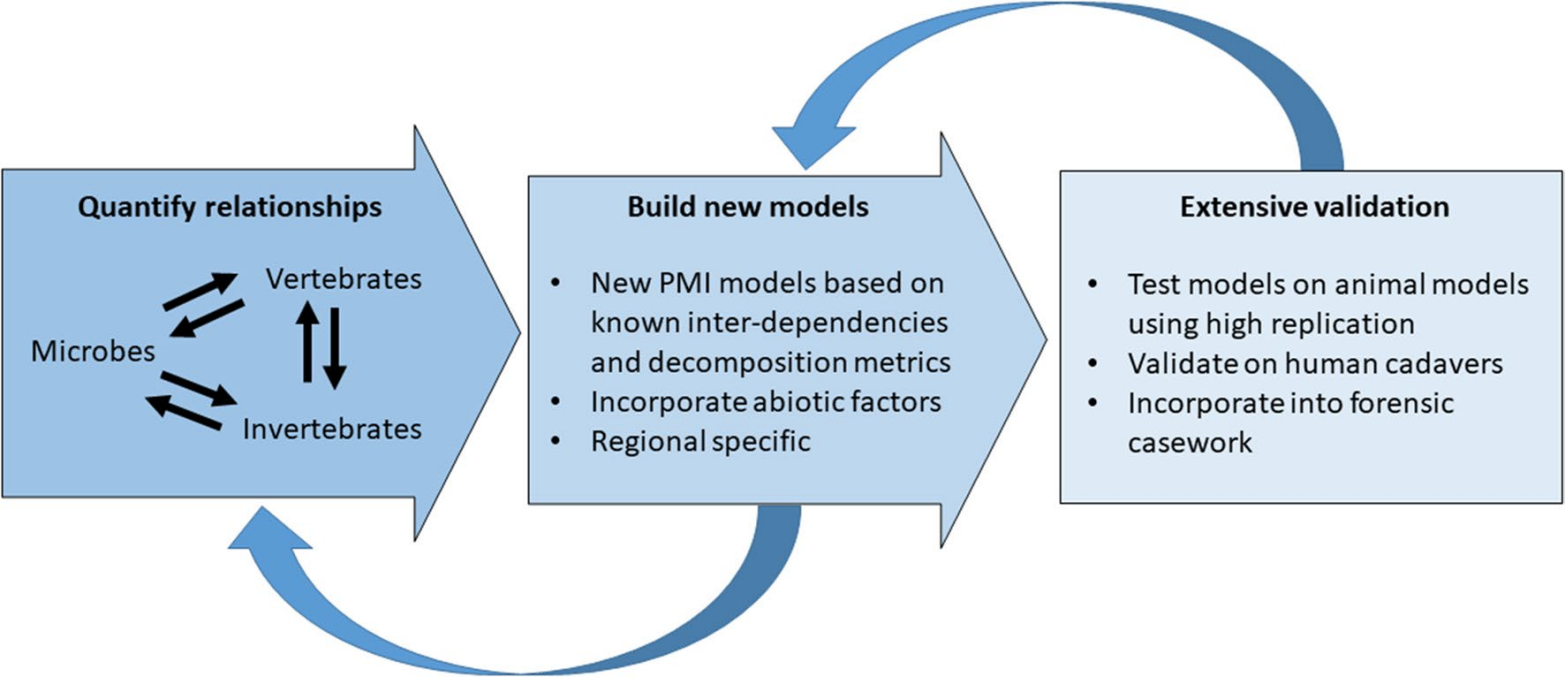
A structured and iterative research approach to using our framework should focus on (i) quantifying fundamental relationships and interdependencies, (ii) building new models based on findings, and (iii) conducting extensive validation of models to forensic casework and PMI estimation. An adaptive approach should also be incorporated at each step (iv), allowing for circling back to reflect on new findings, test new hypotheses, and redevelop relationships and models

At each step, there should be an adaptive component whereby lessons and discoveries enable a step back when necessary to further refine or improve the PMI model. For example, the importance of a particular interaction between two drivers of variation may be discovered when attempting to validate a model, thereby requiring a revaluation of the original model and further consideration of interdependencies (Fig. 6).

## Considerations for application to medicolegal death investigation

Although the current paper focuses on bridging ecological theory and forensic research to improve estimates of PMI, it is important to consider the ultimate goal—assisting medicolegal death investigation. It is crucial to develop a means through which a multidisciplinary framework can be incorporated into the investigative workflow. All medicolegal death investigations are concerned with the primary goals of identifying the decedent, establishing cause and manner of death, and notifying next of kin. In contrast, estimating PMI is not a routine component of an investigation unless it is necessary to help achieve one of the primary goals or assess the reliability of a statement/alibi. Furthermore, it is not necessary to estimate PMI to file a death certificate although, somewhat frustratingly, questions about PMI are the most common questions asked by the bereaved. Bridging this gap between certifying death and providing closure to the bereaved will also benefit from a multidisciplinary framework that is poised to assist with actual medicolegal death investigation.

## Conclusions

New technologies and rapid advances in knowledge create further need to update and validate more sophisticated models of decomposition. Using our framework, models will be able to identify sources of variation in decomposition and provide more reliable and accurate PMI measures. Our framework provides a foundation for undertaking multidisciplinary forensic research, which not only will benefit casework, but also research on understanding the broader role of animal decomposition in ecosystems.

## Data Availability

Data available on reasonable request from the authors.
